# Assessment of heavy metals in farmed shrimp, *Penaeus monodon* sampled from Khulna, Bangladesh: An inimical to food safety aspects

**DOI:** 10.1016/j.heliyon.2021.e06587

**Published:** 2021-03-24

**Authors:** Chinmoy Biswas, Sadia Sarmin Soma, Md. Fazle Rohani, Md. Hamidur Rahman, Abul Bashar, Md. Sazzad Hossain

**Affiliations:** Department of Aquaculture, Bangladesh Agricultural University, Mymensingh, 2202, Bangladesh

**Keywords:** Heavy metals, Food safety, Human health risk assessment, Shrimp (*Penaeus monodon*)

## Abstract

The analytical experiment was executed to present detailed reports on the concentration of heavy metals (nickel, iron, zinc, manganese, chromium, lead, and cadmium) in farmed shrimp, *Penaeus monodon* and its concomitant human health risks upon consumption. A total of 147 farms from six sub-districts of Khulna were selected for sample collection and concentration of heavy metals were determined by Atomic Absorption Spectrometry (AAS) method, following electro-thermal heater digestion. Ni and Cr were found considerably below the detectable limit (BDL) in four sub-districts, while Cr found in shrimps from Rupsa and Paikgacha were far higher than the maximum recommended limit defined by FAO and WHO. The average concentrations of Fe and Mn in all sub-districts crossed the recommendations, whereas average concentrations of Zn, Ni, Cd, and Pb were within the recommendations. Regardless of sampling site, target hazard quotients (THQ) of more than 1 contributed by Fe confirmed higher level of hazard index (HI), indicating potential human health risk. Fortunately, no heavy metal or their additive effect found to offer lifetime potency of carcinogenesis upon consumption of these shrimps. Therefore, probabilistic non-carcinogenic human health risk from Fe contamination necessitates stringent monitoring and controlling of this metal from different sources to farms.

## 1 Introduction

Shrimp (*Penaeus monodon*), rich in protein, minerals, vitamins, antioxidants, essential amino acids, and unsaturated fatty acids [1, 2], is considered as one of the most beneficial shellfish aliments for human consumption. Bangladesh, the 5^th^ ranked aquaculture producing country [3], produces vast amount (0.239 million metric tons in Fiscal Year 2017–18) of shrimp each year. This huge production earns significant amount of foreign currency (503.93 million USD in 2018) [4] by exporting to the global markets, particularly, in the USA, Europe and Japan [5]. Recently, ceiling concern on nutritional and medicinal values of shrimps have made the national consumption ever increased in Bangladesh [6].

Various types of toxicities arisen from the pollutants including heavy metals [6, 7], microplastics [8, 9], pesticides [10, 11] etc. have made the aquatic faunal communities one of the most unvoiced victims. Among the pollutants, heavy metals in aquatic systems are mainly sourced from anthropogenic practices, including agricultural deeds, landfill erosions, embarkation and docking activities, industrial and domestic wastewater as well as natural processes [6, 12, 13]. In general, non-degradable heavy metals even in trace amount can cause toxicities in aquatic ecosystems through assimilation, deposition, or incorporation at a specific concentration into abiotic components and finally, adopting the path of bio-accumulation into aquatic animals [14]. In aquatic ecosystems, food chain is considered as the main pathway of heavy metals accumulation and metals can create human health hazards upon consumption of these contaminated aquatic foods [15].

Although a number of metals are essential for living organisms, some are highly toxic or become toxic at high concentration. Metals such as lead (Pb), tin (Sn), nickel (Ni), cadmium (Cd), and chromium (Cr) are not generally required for metabolic activities. Moreover, trace amount of these heavy metals can cause toxicities to animals [16]. Besides their carcinogenic effects, heavy metals can cause serious problems, such as liver disorders, cardiovascular anomalies, kidney failure and death in case of extreme situation [17, 18]. Crossing the maximum tolerable limits, heavy metal contamination not only constitutes significant human health risks [6, 15] but also possesses several negative effects on natural balances of the ecosystem [19, 20]. Considering these negative impacts and associated health risks, heavy metal contamination is considered as the most dangerous problem in aquatic ecosystems.

Being a top trencherman in aquatic food chain, shrimp is normally more susceptible to the accumulation of heavy metals from different sources including water, sediments, and foods [13]. Human are exposed to heavy metals mainly through foods, including seafoods, though other media like water, air, and soil can contribute largely [21]. Thus, toxicities arising from the heavy metal accumulation avert the health beneficiary aspects of shrimps, while consumers are paying more attention to the food safety issues nowadays. Therefore, determination of heavy metal in widely consumed farmed shrimps with its possible health risk is of prior importance [22]. To mediate human health risk posed by the heavy metal contaminations, FAO and WHO defined the maximum recommended limits for each heavy metal (Table 1). However, these recommended values solely can't measure the probabilistic carcinogenic and non-carcinogenic human health risks. Hence, US Environmental Protection Agency established quantitative frameworks in favor of quantifying potential hazard index (HI) and target cancer risk (TR) posed by heavy metals [23]. In current study, levels of heavy metals (mg/kg) in shrimps from Khulna, a major shrimp producing hotspot of Bangladesh, were determined by Atomic Absorption Spectrometry (AAS) method. Besides comparing with the maximum limits recommended by FAO and WHO [24], probabilistic HI and TR were also enumerated to interpret whether shrimps from the study areas are safe for human consumption or not.

**Table 1:** Maximum recommended limits of heavy metals for human consumption defined by WHO and FAO [1].

Heavy metals	Maximum recommended limits for human consumption
Nickel	1 mg/kg
Iron	100 mg/kg
Zinc	100 mg/kg
Manganese	1 mg/kg
Chromium	0.05 mg/kg
Lead	2 mg/kg
Cadmium	1 mg/kg

## 2 Materials and methods

### 2.1 Study location and ethical approval

**Figure 1:**
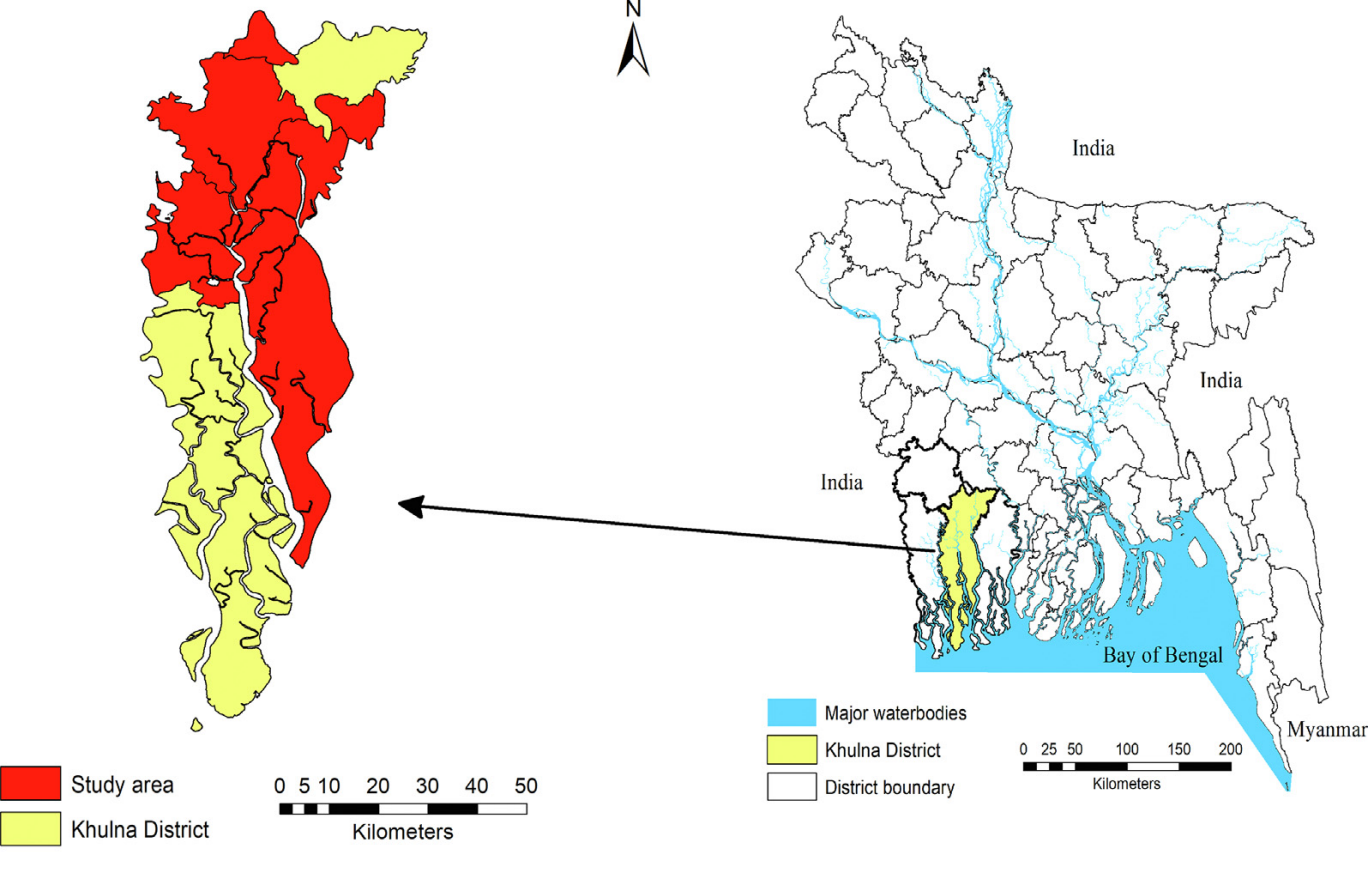
Study area.

Based on outstanding signature in shrimp production, six sub-districts of Khulna, namely Phultola (22.9750°N 89.4583°E), Rupsa (22.8333°N 89.5833°E), Dumuria (22.8083°N 89.4250°E), Paikgacha (22.5889°N 89.3361°E), Batiaghata (22.7417°N 89.5167°E) and Dacope (22.5722°N 89.511°E) were selected for sample collection (Figure 1). Further digestion and analysis were performed in Fish Nutrition Laboratory and Interdisciplinary Institute for Food Security (IIFS) Laboratory of Bangladesh Agricultural University (BAU). The Ethical committee of Bangladesh Agricultural University Research System (BAURES) approved the design and execution of the study.

### 2.2 Sample collection

Samples were collected from 147 extensive farms (24 from Rupsa, 24 from Phultola, 24 from Dacope, 25 from Batiaghata, 30 from Paikgacha and 20 from Dumuria). After washing with distilled water, shrimps were carried to the Fish Nutrition Laboratory for further processing in sealed, labeled and iced condition.

### 2.3 Analysis of heavy metals

#### 2.3.1 Sample preparation

From each sample, approximately 100 g of edible muscle was taken in a clean brown envelope and placed in a hot-air oven to dry at 105 °C for a period of 24 h. After drying, the samples were pulverized with the help of a grinder. Prior to use, all the glass equipment were kept in diluted HNO_3_ for 24 h and then washed with distilled water.

#### 2.3.2 Electro-thermal heater digestion

After treatment with 10 ml HNO_3_ and 5 ml HClO_4_ solution, exactly 1 g from each sample was digested at 80 °C for 30 min in an electro-thermal heater (Model-VELP). The digested samples were cooled and transferred into clean volumetric flasks. Double distilled water was added to make each solution exactly 100 mL. Finally, Whatman Filter paper No. 42 was used to filtrate the solutions before keeping in sealed and labeled plastic bottles.

#### 2.3.3 Blank preparation

Using standard procedure, a blank containing same digestion inputs except sample was prepared to make sure that impurities or contaminations (if any) from the chemicals didn't bias the values [25]. The blank value found through the analysis by AAS was subtracted from each of the sample value to get the true value.

#### 2.3.4 Sample analysis

A flame atomic absorption spectrophotometer (Model Shimadzu AA-7000) was used to determine heavy metals concentration, where acetylene gas and air were used as fuel and oxidizer, respectively. Aspiration of the digested samples was performed using the air acetylene flame. The concentrations of heavy metals were determined with the support of calibration curves relying on Beer Lambert's law [26]. Calibrations by consecutive dilution were achieved using standard solutions as manufacturer's protocol. Determination was based on average values of triplicates for each sample. Absorption wavelengths of 228.0 nm, 217.0 nm, 213.9 nm, 279.5 nm, 232.0, 248.3, and 357.9 were maintained for determination of Cd, Pb, Zn, Mn, Ni, Fe, and Cr, respectively. Detection limit of the spectrophotometer is 0.01 mg/kg and the concentrations below the limit were termed as BDL (Below detectable limit).

### 2.4 Data processing

After determination of heavy metals concentration, all recorded data were collected and processed using Microsoft Excel (MS 2010) to produce graphical and tabular presentation comparing with maximum recommended limits.

### 2.5 Human health risk assessment

To assess the potential health risk, target hazard quotient (THQ) for each heavy metal was calculated adopting the scientific formula (Eq. (1)) established by USEPA [27].

(1) $$\text{THQ}=\frac{{\text{E}}_{\text{D}}\times {\text{F}}_{\text{IR}}\times {\text{E}}_{\text{F}}\times {\text{C}}_{\text{i}}}{{\text{R}}_{\text{FD}}\times {\text{W}}_{\text{AB}}\times {\text{T}}_{\text{A}}}\times 10^{-3}$$

where,

E_D_ = Exposure duration (Average life span, 72.32 years)

F_IR_ = Daily ingestion rate (2.43 gm/person/day, determined from an online based survey with 5 thousand respondents throughout the country)

E_F_ = Exposure frequency (365 days/year)

C_i_ = Concentration of respective heavy metal (mg/kg)

R_FD_ = The reference oral dose in mg/kg/day (0.001 for Cd, 0.004 for Pb, 1.5 for Cr, 0.3 for Zn, 0.02 for Ni, 0.007 for Fe, 0.14 for Mn according to USEPA [27])

W_AB_ = Average body weight for an adult consumer (54.6 kg for Bangladesh, according to the online based survey)

T_A_ = Average exposure time, calculated as E_D_ × E_F_

The overall hazard index (HI) was calculated using following formula (Eq. (2)) according to USEPA [27].

(2) $$HI=THQ_{Fe}+THQ_{Zn}+THQ_{Ni}+THQ_{Mn}+THQ_{Cd}+THQ_{Pb}+THQ_{Cr}$$

Among the analyzed heavy metals, Cd, Cr, Ni, Pb were considered as potent carcinogens. Target cancer risk (TR) posed by the determined heavy metals was calculated according following formula (Eq. (3)) [28]:

(3) $$\text{TR}=\frac{{\text{E}}_{\text{D}}\times {\text{F}}_{\text{IR}}\times {\text{E}}_{\text{F}}\times {\text{C}}_{\text{i}}\times {\text{C}}_{SF}}{{\text{W}}_{\text{AB}}\times {\text{T}}_{\text{A}}}\times 10^{-3}$$

The values of cancer slope factors (C_SF_) were adopted from USEPA [27] (for Cd (6.3 mg/kg/day) and Pb (0.0085 mg/kg/day)) and Zeng et al. [29] (for Ni (0.91 mg/kg/day) and Cr (0.5 mg/kg/day)).

## 3 Results

### 3.1 Heavy metals concentration

The overall finding of metal concentrations found from analyzed shrimp samples is presented in Table 2.

**Table 2:** Average concentration of heavy metals (Ni, Fe, Zn, Mn, Cr, Pb, Cd) in shrimps collected from six sub-districts of Khulna.

Sub-districts	Concentrations of heavy metals (mg/Kg)						
	Ni	Fe	Zn	Mn	Cr	Pb	Cd
Rupsa	BDL^1^	358.990 ± 52.139	82.280 ± 3.851	17.250 ± 3.397	0.084 ± 0.022	0.691 ± 0.074	0.049 ± 0.001
Phultola	BDL	331.130 ± 57.795	84.103 ± 6.589	26.450 ± 9.295	BDL	0.502 ± 0.036	0.044 ± 0.002
Dacope	BDL	310.910 ± 22.459	73.368 ± 9.976	6.550 ± 2.576	BDL	0.418 ± 0.027	0.041 ± 0.006
Batiaghata	BDL	184.084 ± 32.636	74.864 ± 14.088	37.870 ± 11.247	BDL	0.362 ± 0.022	0.040 ± 0.004
Paikgacha	0.080 ± 0.515	219.888 ± 20.795	80.678 ± 6.443	35.220 ± 11.369	0.235 ± 0.071	0.361 ± 0.019	0.041 ± 0.001
Dumuria	0.042 ± 0.013	211.012 ± 37.813	74.464 ± 4.169	34.780 ± 8.877	BDL	0.354 ± 0.040	0.041 ± 0.003

1 Below detectable level.

#### 3.1.1 Ni concentrations

**Figure 2:**
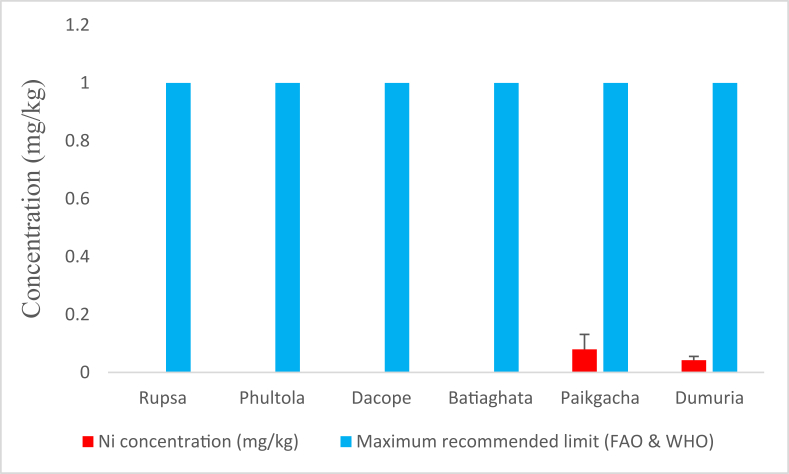
Average nickel (Ni) concentrations (mg/kg) in shrimp of Khulna district compared with maximum recommended limit.

Average Ni concentrations in the shrimps of Paikgacha and Dumuria were found 0.080 (±0.052) mg/kg and 0.042 (±0.013) mg/kg, respectively (Figure 2). Other sub-districts were reported to have Ni below detectable level (BDL). However, recorded Ni concentration didn't cross the recommended limits [24].

#### 3.1.2 Fe concentrations

**Figure 3:**
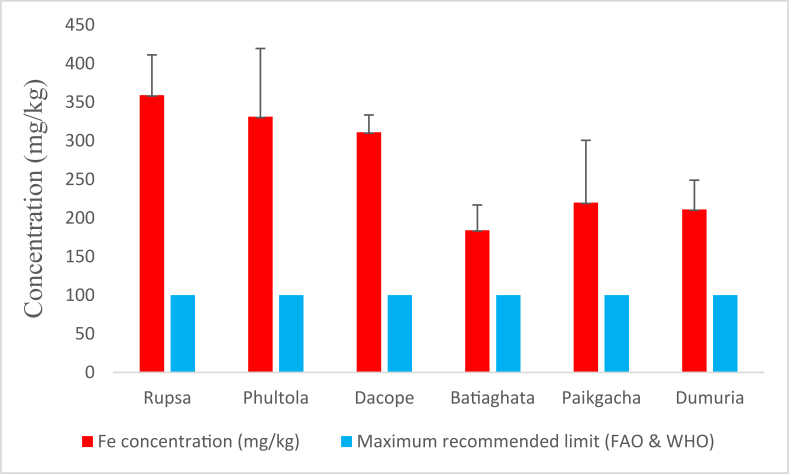
Average iron (Fe) concentrations (mg/kg) in shrimp of Khulna district compared with maximum recommended limit.

The highest average concentration of Fe (358.995 ± 52.139 mg/kg) was observed in the shrimps collected from Rupsa whereas the lowest average (184.084 ± 32.636 mg/kg) from Batiaghata (Figure 3). However, the average Fe concentrations determined from all sampling sites exceeded the maximum recommended limit [24].

#### 3.1.3 Zn concentrations

**Figure 4:**
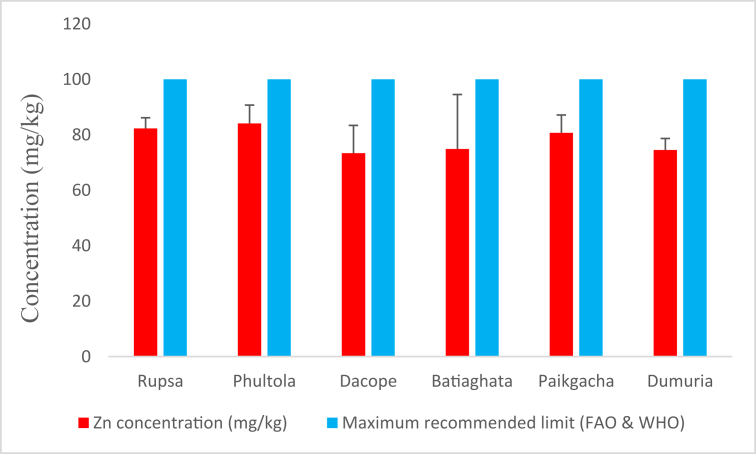
Average zinc (Zn) concentrations (mg/kg) in shrimp of Khulna district compared with maximum recommended limit.

The highest average level of Zn (84.103 ± 6.589 mg/kg) was observed in the shrimps of Phultola, while the shrimps from Dacope offered the lowest average concentration (73.368 ± 9.976 mg/kg) (Figure 4). However, average Zn concentrations found in shrimps from different sub-districts were lower than the recommendation [24].

#### 3.1.4 Mn concentrations

**Figure 5:**
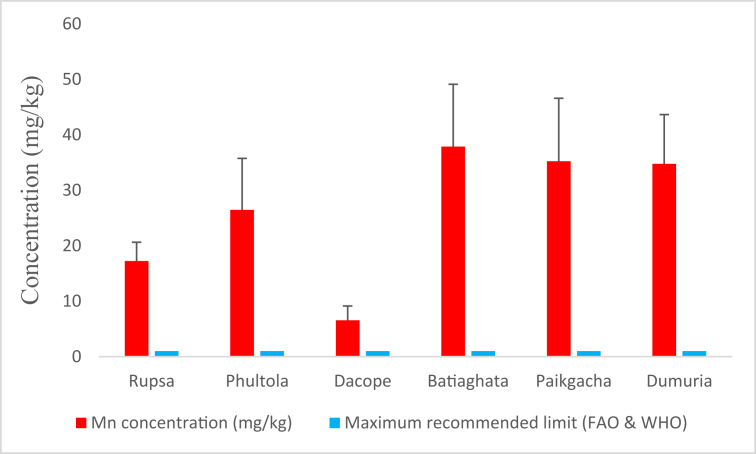
Average manganese (Mn) concentrations (mg/kg) in shrimp of Khulna region compared with maximum recommended limit.

Average Mn concentration was found to be the highest (37.87 ± 11.247 mg/kg) in Batiaghata and the lowest (6.550 ± 2.576) in Dacope (Figure 5). The average concentrations found in all sub-districts were far higher than the maximum recommended level of FAO and WHO [24].

#### 3.1.5 Cr concentrations

**Figure 6:**
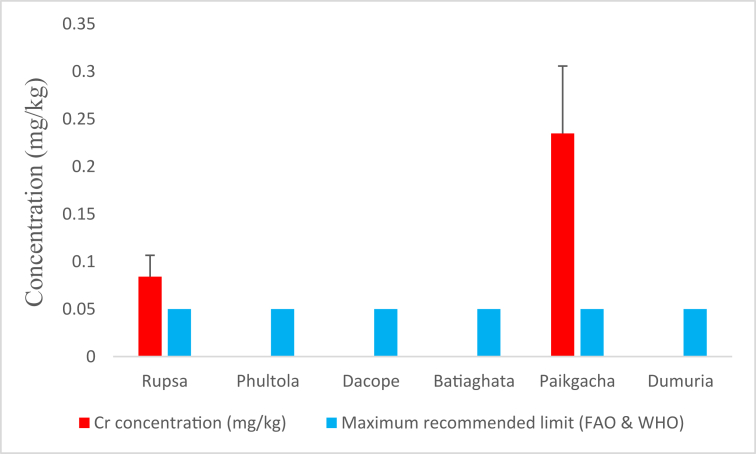
Average chromium (Cr) concentrations (mg/kg) in shrimp of Khulna district compared with maximum recommended limit.

Shrimps of Rupsa and Paikgacha were suffered from Cr contamination with an average concentration of 0.084 (±0.022) and 0.235 (±0.071) mg/kg, respectively where both values crossed the recommendation. Average chromium concentrations in the rest sub-districts were below detectable limit (Figure 6).

#### 3.1.6 Pb concentrations

**Figure 7:**
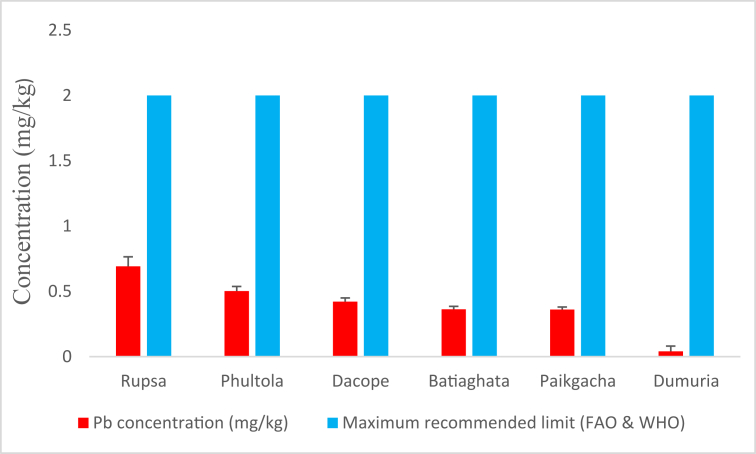
Lead (Pb) concentrations (mg/kg) in shrimp of Khulna district compared with maximum recommended limit.

The maximum average concentration of Pb (0.691 ± 0.074 mg/kg) was reported from Rupsa, while the minimum (0.354 ± 0.040 mg/kg) from Dumuria (Figure 7). However, values recorded from all sampling sites were far below than the maximum recommended value [24].

#### 3.1.7 Cd concentrations

**Figure 8:**
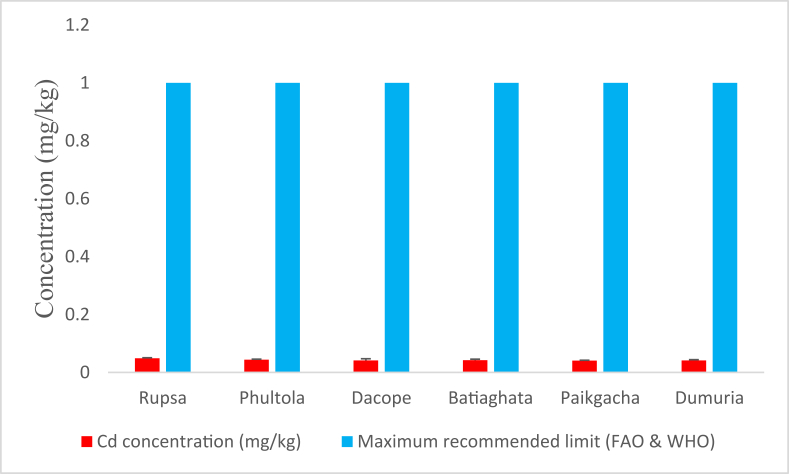
Average cadmium (Cd) concentrations (mg/kg) in shrimp of Khulna district compared with maximum recommended limit.

The highest average Cd concentration (0.0491 ± 0.001 mg/kg) was found in shrimps from Rupsa and somewhat similar results were reported from other sub-districts (Figure 8). However, no determined concentration crossed the maximum recommended limits defined by FAO and WHO [24].

### 3.2 Human health risk assessment

Though the determined concentrations of Mn and Cr (in Rupsa and Paikgacha) crossed the recommended values, THQ values solely for Fe were higher than 1 in all sub-districts (Table 3). This made the HI elevated over 1 in all sites. From spatial consideration, shrimps of Rupsa possessed highest level of non-carcinogenic health risk, whereas Batiaghata did the lowest. No determined TR value, nor their additive effect (TRt) exceeded the recommended value (<10^−4^). However, Phultola presented the highest TR values contributed largely by Cd, whereas Dumuria did the lowest.

**Table 3:** Calculated target hazard quotients (THQ), hazard index (HI) and target cancer risk (TR) for each heavy metal traced from the shrimp samples.

Risk indexes	Rupsa	Phultola	Dacope	Batiaghata	Paikgacha	Dumuria
Target hazard quotients (THQ)						
THQ_Ni_	NA	NA	NA	NA	3.24E-06	9.35E-05
THQ_Fe_	**2.282**	**2.105**	**1.977**	**1.170**	**1.398**	**1.341**
THQ_Zn_	0.012	0.013	0.011	0.011	0.012	0.011
THQ_Mn_	0.006	0.008	0.002	0.012	0.011	0.011
THQ_Cr_	2.49E-06	NA	NA	NA	6.96E-06	NA
THQ_Pb_	0.008	0.006	0.005	0.004	0.004	0.004
THQ_Cd_	0.002	0.006	0.002	0.002	0.002	0.002
HI	**2.310**	**2.137**	**1.996**	**1.199**	**1.427**	**1.370**
Target cancer risk (TR)						
TR_Ni_	NA	NA	NA	NA	3.24E-06	1.701E-06
TR_Cd_	1.38E-05	3.52E-05	1.16E-05	1.18E-05	1.15E-05	1.15E-05
TR_Cr_	1.87E-06	NA	NA	NA	5.22E-06	NA
TR_Pb_	2.62E-07	1.90E-07	1.59E-07	1.37E-07	1.37E-07	1.34E-07
TRt	1.59E-05	3.54E-05	1.18E-05	1.19E-05	2.01E-05	1.34E-05

Values Exceeded recommendation are indicated as bold.

## 4 Discussion

This study was undertaken to determine heavy metal concentrations in shrimps collected from six sub-districts of Khulna and to assess the probabilistic human health impacts upon consumption. According to afore mentioned findings, Figure 2 points that Ni was considerably low (below detectable limit) in shrimps of Rupsa, Phultola, Dacope, and Batiaghata. Though traced in shrimps of Paikgacha and Dumuria, did not cross the maximum recommended level (1 mg/kg) of Ni [24] and also the value (2.5 mg/kg) determined by Vinodhini and Narayana [30] in *Cyprinus carpio*. Another study by Rejomon et al. [31] which found Ni concentrations to vary between 12.12 and 13.92 mg/kg in the marine fishes from southwest coast of India disagrees with our findings. This may be because, Ni concentrations and their sensitivity in water fluctuates with species, abiotic components including salinity of water, location and industrial process around the water [32]. However, an average THQ lower than 1 for Ni in our study areas suggests no human health concern from Ni consideration.

Figure 3 shows that the Fe concentrations in all shrimps were far higher than the recommended limit [24]. The results also crossed all other findings of 36.211 mg/kg [33] and 6.570 mg/kg [34], of 27.22 mg/kg [35], and of 8.819 mg/kg [36] in fishes from Turkey, Cambodia, and Italy, respectively. Among heavy metals, Fe concentrations were highest in all shrimp samples (Table 2). THQ_Fe_ values more than 1 confirm that shrimps from the study areas were not safe for human consumption. Generally, this 2^nd^ most abundant metal [37] is accumulated in shrimps from the feed fed, ground water contamination from mining, and industrial effluents etc. Fe is necessary for binding proteins, activation of coenzymes, and other metabolic activities in human [38]. But a number of detrimental effects are experienced due to high level of Fe when it fails to bind proteins and thus unbound Fe become erosive to the gastrointestinal tracts [39].

The observations on Zn are similar to various studies [39, 40, 41, 42, 43], but higher than the concentrations found in fishes from eastern Taiwan [44], Malaysia [45], Turkey [46], and lower from south west coast of India [31], Indonesia [47] and Iran [48, 49]. However, average THQ_Zn_ values in all sampling sites were within the recommendation. Thus, the study can propose that shrimps from these areas were not harmful from the consideration of Zn contamination. On contrary, this low level of Zn, possibly sourced from feedstuff, water and sediments may accelerate the metabolic process of cultured shrimp to favor the growth [50].

Mn concentrations found in current study are in proximity with the previous studies undertaken by Yilmaz [12] and Abu Hilal and Ismail [51]. This metal, running into water from pharmaceutical, industrial, and agricultural sources, can cause gastro-intestinal and neurological abnormalities to human. Besides, long term exposure of this metal may also cause Parkinson, lung embolism, cancer, thyroid and other abnormalities [52]. Though the concentrations, far higher than the certified level of WHO and FAO, recommend great concern regarding the consumption of shrimps from these areas, tolerable THQMn values allow the Mn concentrations and infer no possible.

Our findings regarding Cr concentrations agree with the results obtained from a study carried out in the Bangsi river of Bangladesh [53], but differ from the results reported from the Kabdak river of Satkhira, Bangladesh [54]. Articulation revealed that concentrations of Cr in shell (1.03 mg/kg) was much higher than in tissue (0.68 mg/kg) of black tiger shrimps of Batiaghata, while similar concentration (0.14 mg/kg) was observed in both tissue and shell for freshwater prawn of the Bhairab river of Bangladesh [6]. However, both findings exceed our determined values. As feed inputs, poultry droppings and tannery wastes are frequently used in shrimp farms in Bangladesh. These Cr-rich wastes cause Cr uptake into shrimp body and this made the understanding behind the Cr concentrations in shrimps of Rupsa and Paikgacha that crossed the maximum recommended limit of WHO and FAO. Cr uptake in human body for a long time can cause disruption of cellular integrity and functions by damaging protein and lipid membrane [55, 56]. Fortunately, THQ_Cr_ values are very negligible and confirm no potent human health risk from Cr consideration.

Ahmed et al. [57] and Sarkar et al. [6] documented Pb concentrations of 0.51 mg/kg in freshwater prawn from the Buriganga river and 0.52–1.16 mg/kg in shrimps from Khulna-Satkhira region of Bangladesh, respectively, which are somewhat close to our findings. Pb can cause renal failure and liver damage [58] upon consumption of Pb contaminated foods and prolonged exposure may lead to mental retardation, comma, and even death in severe cases [18]. However, far lower concentration than the recommendation and lower average THQ_Pb_ values in all sub-districts avoid these human health risks.

WHO and FAO defined 1 mg/kg as the maximum recommended limit for Cd while 0.05 mg/kg and 0.5 mg/kg were defined by the European Community legislation [59] and Codex Committee on Food Additives and Contaminants [60], respectively. However, the average Cd concentrations determined in the current study were far below from the recommended level by FAO and WHO; and very close to the European Union recommendation. Our result on Cd concentrations agrees with the finding where authors determined Cd level of 0.05–0.13 mg/kg in shrimps of Khulna- Satkhira region [6]. however, the result for shellfish (1.51 mg/kg) from the Buriganga river [57] counters our findings. Renal and hepatic dysfunctions may be accelerated by high dose Cd exposure while long term exposure may obstruct bone formation, hypertensions, tumors and even cancer in urinary bladder [6, 61]. Nevertheless, lower THQ_Cd_ values in all shrimps sampled from Khulna region deny these health risks posed by cadmium.

An HI index of more than 1 recommends possible human health risk. Though no other metals did, iron contributed to a THQ more than 1 and made the hazard index far higher than the recommendation. From induvial metal perspective, though THQ calculated from Ni, Zn, Mn, Pb, Cr, Cd concentrations complied the human health safety issues, Fe threatens the consumption of shrimps from these areas. Providentially, adverse effects of iron for human health are not so serious like other heavy metals and comprehensive actions regarding minification of its availability in farms can improve the shrimp's quality.

Target cancer risk values augur the lifetime potency of carcinogen(s) [22] and values greater than 10^−4^ are considered to exert potential carcinogenic risks [23]. Recorded TR values for Ni, Cr, Cd and Pb, ranging from 10^−7^ to 10^−5^, are considered acceptable. Cumulative target cancer risk values (TRt) in all sub-districts suggest no potential risk of carcinogenesis from these shrimps.

## 5 Conclusion

The study revealed that the average concentrations of Fe, Mn, and Cr (in Rupsa and Paikgacha) in shrimps from Khulna were considerably higher than the maximum recommended limits. Target hazard quotients of Fe made the hazard indices more than 1 in all sub-districts. However, risk of carcinogenesis posed by Ni, Cr, Pb, and Cd were within the acceptable range. This can wrap a conclusion that the shrimps from these areas can cause non-carcinogenic harm to human upon consumption. Therefore, to ensure the food safety aspects from detrimental consequences of heavy metal contaminations, execution of standards in all steps of shrimp production is obligatory.

## Declarations

### Author contribution statement

Chinmoy Biswas: Conceived and designed the experiments; Performed the experiments.

Sadia Sarmin Soma, Hamidur Rahman: Performed the experiments.

Fazle Rohani, Abul Bashar: Analyzed and interpreted the data; Wrote the paper.

Sazzad Hossain: Conceived and designed the experiments; Contributed reagents, materials, analysis tools or data.

### Funding statement

This work was supported by BAS-USDA (2017/171/BAS-USDA).

### Data availability statement

Data will be made available on request.

### Declaration of interests statement

The authors declare no conflict of interest.

### Additional information

No additional information is available for this paper.
